# Induced pluripotent stem cell-derived tenocyte-like cells promote the regeneration of injured tendons in mice

**DOI:** 10.1038/s41598-020-61063-6

**Published:** 2020-03-04

**Authors:** Shingo Komura, Takashi Satake, Atsushi Goto, Hitomi Aoki, Hirofumi Shibata, Kenji Ito, Akihiro Hirakawa, Yasuhiro Yamada, Haruhiko Akiyama

**Affiliations:** 1 Department of Orthopaedic Surgery, Gifu University Graduate School of Medicine, Gifu, 501-1194 Japan; 2 Department of Tissue and Organ Development, Regeneration, and Advanced Medical Science, Gifu University Graduate School of Medicine, Gifu, 501-1194 Japan; 3 Laboratory of Stem Cell Oncology, Department of Life Science Frontiers, Center for iPS Cell Research and Application (CiRA), Kyoto University, Kyoto, 606-8507 Japan; 4 Division of Stem Cell Pathology, Center for Experimental Medicine and Systems Biology, Institute of Medical Science, University of Tokyo, Tokyo, 108-8639 Japan

**Keywords:** Pluripotent stem cells, Regeneration

## Abstract

Tendons are dense fibrous structures that attach muscles to bones. Healing of tendon injuries is a clinical challenge owing to poor regenerative potential and scarring. Here, we created reporter mice that express EGFP, driven by the promoter of the tendon-specific Scleraxis (*Scx*) transcription-factor gene; we then generated induced pluripotent stem cells (iPSCs) from these mice. Utilising these fluorescently labelled iPSCs, we developed a tenogenic differentiation protocol. The iPSC-derived EGFP-positive cells exhibited elevated expression of tendon-specific genes, including *Scx*, *Mohawk*, *Tenomodulin*, and *Fibromodulin*, indicating that they have tenocyte-like properties. Finally, we demonstrated that these cells promoted tendon regeneration in mice after transplantation into injured tendons reducing scar formation via paracrine effect. Our data demonstrate that the tenogenic differentiation protocol successfully provided functional cells from iPSCs. We propose that pluripotent stem cell-based therapy using this protocol will provide an effective therapeutic approach for tendon injuries.

## Introduction

Tendons are fibrous connective tissues that attach muscles to bones. Tendon injuries and tendinopathies caused by overuse or age-related degeneration are common problems in adult patients, constituting approximately 30% of musculoskeletal diseases^1^. Tendon tissue has a slow metabolism and tolerates hypoxia; however, it requires a long period to reacquire sufficient strength after injury due to poor regenerative potential caused by its hypocellularity and hypovascularity^2,3^. Recent studies have demonstrated that injured tendons in adult mice do not recover by building normal tendon tissue, but by building scar tissue produced by myofibroblasts^4^. Scar tissue has lower tensile strength than normal tendon tissue; therefore, physiological healing of tendon disorders in adult patients remains a significant medical challenge.

Several potential approaches for tendon regeneration have been developed, including pharmacological, biomaterial, and cell-transplantation therapies using stem/progenitor cells^1,2^. Stem-cell therapy can exploit multiple sources, including mesenchymal stem cells (MSCs), adipose-derived stem cells (ADSCs), tendon stem/progenitor cells (TSPCs), embryonic stem cells (ESCs), and induced pluripotent stem cells (iPSCs)^2,3,5–9^. Since the discovery of iPSCs^10,11^, the differentiation of many cell types has been induced from them, via well-established protocols^12–14^.

Although there have been reports of several tenogenic differentiation protocols from pluripotent stem cells (iPSCs/ESCs) using transforming growth factor (TGF)-β3 and three-dimensional culture^15,16^, bone morphogenic protein (BMP) 12/13 and ascorbic acid^17^, and well-aligned, chitosan-based ultrafine fibers^18^, none have described the isolation of tenogenic cells with measurements of induction efficiency. Moreover, only a few studies have addressed therapeutic validity *in vivo*^18^.

A basic helix-loop-helix transcription factor, Scleraxis (*Scx*) is a tendon-specific marker, expressed in tenoblasts and tenocytes during embryogenesis and into adulthood^19,20^. *Scx-GFP* transgenic mice have been created^21,22^ and used to investigate mechanisms of tendon development and the biological responses of tenocytes to cytokines and mechanical stress^6,23–25^. We reasoned that this tendon-specific reporter system could be of great value in the development of a tenogenic differentiation protocol from pluripotent stem cells, which could then be exploited for cell-based therapy for tendon injuries. We therefore created a new, knock-in *Scx-EGFP* allele in mice. In this study, we used these tendon-specific reporter mice to produce iPSCs with the *Scx-EGFP* reporter system. We exploited the reporter system to develop a tenogenic differentiation protocol from iPSCs. Upon transplantation of the differentiated cells into injured tendons, they promoted tendon regeneration in mice.

## Results

### *Scx-EGFP* knock-in mice

**Figure 1:**
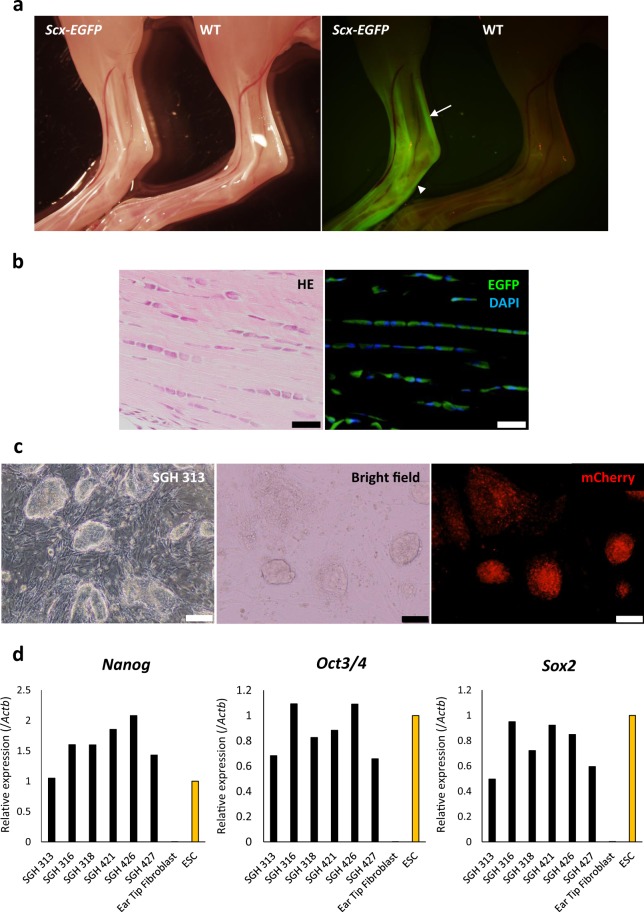
Generation of *Scx-EGFP* reporter mice and establishment of iPS cell lines. (**a**) Bright field and fluorescence images of the ankles of 3-wk-old *Scx-EGFP* homozygous (left) and control littermate homozygous wild-type (WT, right) mice. The *Scx-EGFP* homozygous mouse exhibited bright EGFP signals in tendons around the ankle including Achilles tendon and plantar fascia, whereas wild-type mouse did not. White arrow indicates Achilles tendon and white arrow head indicates the plantar fascia. (**b**) Histology of an Achilles tendon from a 3-wk-old *Scx-EGFP* homozygous mouse. Top, hematoxylin/eosin; bottom, EGFP signal (green) detected in tenocytes. Scale bars, 20 µm. (**c**) Micrographs of iPSCs derived from *Scx-EGFP* ear-tip fibroblasts. iPSCs were labelled with mCherry. Cells from clone SGH 313 are shown. Scale bar, 100 μm. (**d**) Expression of pluripotency-related genes (*Nanog*, endogenous *Oct3/4*, and endogenous *Sox2*) in SGH iPS cell lines was equivalent to that of ES cells, as determined by qRT-PCR. The expression level in ES cells was set to 1. Data are presented as the mean of three technical replicates.

We utilised the Red/ET recombination system, by inserting *IRES-EGFP* sequences into the *Scx* coding and regulatory regions after the stop codon of the *Scx* gene in a bacterial artificial chromosome (BAC) (Supplementary Fig. 1a). Targeting vectors were excised from the BAC and electroporated into ESCs. Positive clones were confirmed by southern blots (Supplementary Fig. 1b). Chimeric mice were generated from the positive clones to obtain *Scx-EGFP* mice through germline transmission, which showed tendon- and ligament-specific EGFP expression (Fig. 1a,b, and Supplementary Fig. 2).

To produce homozygous *Scx-EGFP* mice, we intercrossed heterozygous mice; however, we obtained no homozygotes. The reason for this may be the *Scx* gene is located in the intronic region of the block of proliferation 1 (*Bop1*) gene, which is involved in ribosome biogenesis and chromosomal segregation^26^. A previous study reported that artificial promoters in a drug-selection cassette affected *Bop1* regulation and that their deletion allowed the successful generation of *Scx* homozygous knock-in mice^27^. Therefore, to delete the drug selection cassette, *Scx-EGFP* mice were crossed with *Dre* recombinase-expressing mice^28^ (Supplementary Fig. 3a). Mice homozygous for the *Scx-EGFP* allele lacking the drug selection cassette were viable and normal in size, and had normal reproductive potential (Supplementary Fig. 3b,c).

### Establishment of iPSCs from *Scx-EGFP* fibroblasts

Recent research using the existing *Scx-GFP* transgenic line has demonstrated that neonatal tendons could physiologically heal after injury, whereas adult tendons could not^4^. Similarly, when we cut the Achilles tendons of our *Scx-EGFP* neonates (7 d) and adults (4 mo), their healing was completely consistent with that observed by Howell *et al*. Physiological tendon healing with EGFP signal was observed in neonatal mice at 1 mo after injury, whereas chondrometaplasia at tendon stumps and alpha smooth-muscle actin (αSMA)-expressing, myofibroblast-induced scar healing with no EGFP signal were observed in adult mice (Supplementary Fig. 4a,b). These data suggest that healing of injured adult tendons will benefit from regenerative therapy.

We therefore induced the differentiation of tenocytes from iPSCs from our knock-in line, using EGFP fluorescence as a marker for successful induction. First, we reprogrammed ear-tip fibroblasts from *Scx-EGFP* homozygotes by reprogramming four factors (*OCT4*, *SOX2*, *KLF4*, and *MYCL*) and established three iPSC clones (SGH #3-1, #4-1, and #4-2; Supplementary Fig. 5a). Clones SGH #3-1 and #4-2 were then transfected with a construct expressing mCherry driven by the CAG promoter, producing 6 mCherry-expressing iPSC lines (SGH 313, 316, 318, 421, 426, and 427; Fig. 1c).

Real-time PCR showed that these lines expressed the pluripotency-related genes *Nanog*, *Oct3/4*, and *Sox2* at levels comparable to those in murine ESCs (Fig. 1d). Moreover, silencing of expression of the four exogenous factors was confirmed in mCherry-expressing iPSC lines although they contained multiple retrovirus integrations (Supplementary Fig. 5b,c). Upon subcutaneous transplantation of these iPSC lines into immunocompromised mice, teratoma formation was confirmed (Supplementary Fig. 5d,e). These data indicate successful induction of iPSCs with the *Scx-EGFP* reporter system. Lines SGH 313 and 427 that were brightly and ubiquitously labelled with mCherry were utilised in the experiments described below.

### Induction of *EGFP*-positive, tenocyte-like cells from iPSCs

During embryonic development, paraxial mesoderm generates somites that further differentiate into the following four compartments: myotome, dermatome, sclerotome and syndetome. Tendon progenitors develop from the syndetome, which originates from the dorsolateral edge of the sclerotome in response to fibroblast growth factor (FGF) signalling from the adjacent myotome^19^. FGF and transforming growth factor-β (TGF-β) signalling is essential for tendon development and induction of tendon-specific transcription factors and extracellular matrix genes in tenocytes^29,30^. Bone morphologic protein (BMP) signalling has a negative effect on tendon development^20^.

**Figure 2:**
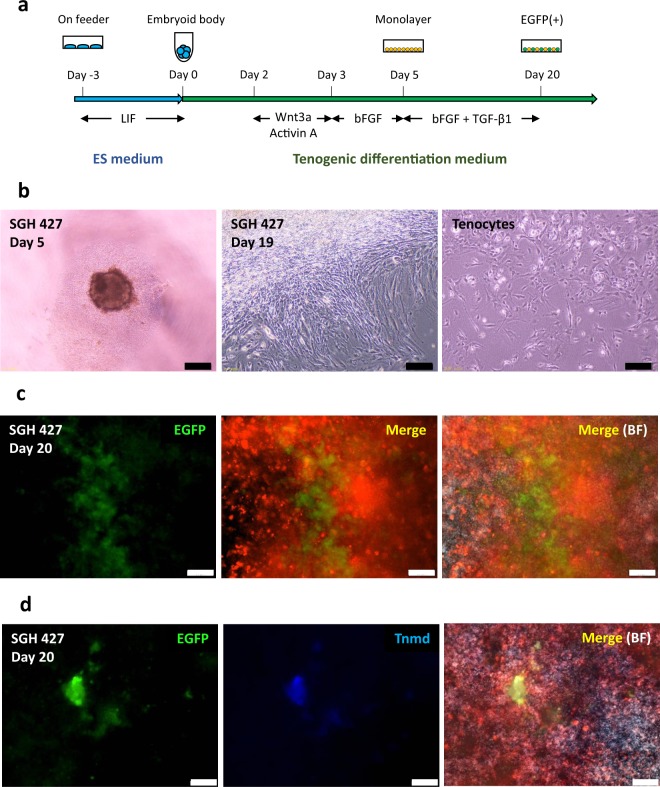
Induction of tenocyte-like cells from iPSCs. (**a**) Schematic of tenogenic differentiation protocol. (**b**) Micrographs of differentiated cells (clone SGH427). Left, day 5; middle, day 19; and right, tenocytes derived from Achilles tendons in *Scx-EGFP* mice (passage 1). Scale bar, 200 µm. (**c**) Fluorescence micrographs of iPSC-derived differentiated cells (clone SGH 427) on day 20, showing that a population of mCherry-labelled cells expresses EGFP. Merge, EGFP and mCherry; BF, bright field. Scale bar, 50 μm. (**d**) Immunocytochemistry of iPSC-derived differentiated cells (clone SGH 427) on day 20. Detection of the tendon-specific marker Tnmd on day 20 after tenogenic differentiation. Scale bar, 50 μm. Merge, EGFP, Tnmd and mCherry; BF, bright field.

By mimicking tendon development and differentiation in embryogenesis, we designed a tenocyte-induction protocol to investigate whether iPSC-derived tenocytes can improve healing in injured adult tendons (Fig. 2a). First, Wnt3a- and Activin A-induced differentiation was started by embryoid body (EB) formation^31^. On day 3, EBs were cultured in basic FGF (bFGF)-containing medium for 2 d. Real-time polymerase chain reaction (PCR) showed Wnt3a and Activin A induced *Gsc*-expressing mesendoderm cells, and followed by bFGF treatment resulted in a decrease in endodermal marker (*Foxa2*) and increase in paraxial mesoderm marker (*Tcf15*) and somite marker (*Nkx3.2* and *Meox1*), indicating mesodermal EB differentiation (Supplementary Fig. 6a)^32^. On day 5, mesodermal EBs were reseeded and tenogenic differentiation was induced by the addition of TGF-β1 and bFGF (Fig. 2b). Expanding cells mainly showed spindle-shaped morphology resembling tenocytes (Fig. 2b). EGFP-expressing cells emerged in the expanding colonies 7 d after reseeding (Fig. 2c). Immunocytochemistry showed that EGFP-expressing cells also expressed Tenomodulin (Tnmd), a tenocyte-specific extracellular-matrix protein (Fig. 2d).

**Figure 3:**
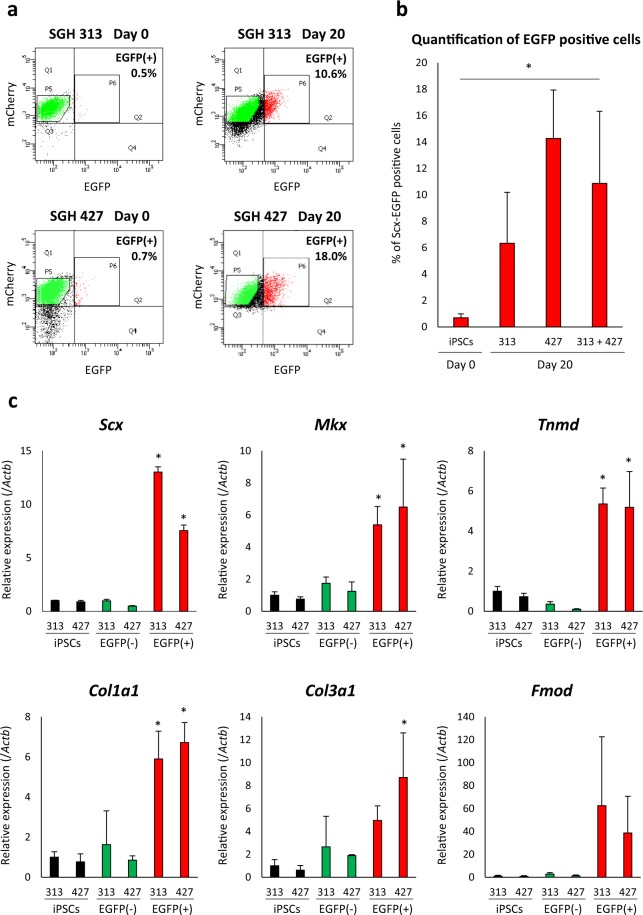
iPSC-derived EGFP-positive cells express tenogenic differentiation markers. (**a**) Flow cytometry of differentiated tenogenic cells. On day 0 (negative control), undifferentiated iPSCs expressed minimal EGFP (0.5% positive in clone SGH 313, 0.7% in SGH 427). On day 20, 10.6% and 18.0% of differentiated cells expressed EGFP in SGH 313 and SGH 427, respectively. (**b**) Tenogenic differentiation efficiency. Repeated FACS experiments were performed for differentiated SGH 313 on day 20 (three independent experiments), differentiated SGH 427 on day 20 (four independent experiments), and undifferentiated iPSC (SGH 313 and SGH 427) on day 0 (five independent experiments). Statistical analysis could not be performed due to less than five experiments in each line; however, when the results from SGH 313 and SGH 427 were combined (n = 7), our differentiation protocol significantly induced EGFP-positive cells from iPSCs. Mean ± SD are shown and Mann–Whitney U test was used to compare between undifferentiated iPSC on day 0 and differentiated SGH 313 and SGH 427 on day 20. Asterisks indicate statistical significance (*P* < 0.05). (**c**) Increased expression of tenogenic differentiation-related genes after differentiation. The mean ± SD (three technical replicates per n; n = 3 biological replicates)) are shown with the expression level of SGH 313 (iPSCs) set to 1. Black bars, iPSCs; green bars, FACS-sorted EGFP-negative cells at day 20; and red bars, FACS-sorted EGFP-positive cells at day 20. Mann–Whitney U test was used to compare between 313 EGFP-negative and -positive, and between 427 EGFP-negative and –positive cells. Asterisks indicate statistical significance (*P* < 0.05).

On day 20 after tenocyte induction, we performed fluorescence-activated cell sorting (FACS) to enrich EGFP-positive cells (Fig. 3a). The mean % of FACS-sorted EGFP-positive cells at day 20 was 6.3% (range; 4.1 to 10.8) for SGH 313 and 14.3% (range; 10.3 to 18.0) for SGH 427 (overall mean; 10.9%), whereas that of undifferentiated iPSC at day 0 (SGH 313 and SGH 427) was 0.7% (range; 0.5–1.2) (Fig. 3b). FACS-sorted EGFP-positive cells showed elevated the expression of the tendon-specific transcription factors *Scx* and *Mkx*, as well as the extracellular matrix genes *Tnmd*, *Col1a1*, *Col3a1*, and *Fmod*. We observed decreased expression of the pluripotency-related genes *Nanog*, *Oct3/4*, and *Sox2* (Fig. 3c and Supplementary Fig. 6b). We also showed our protocol induced the expression of tendon-specific transcription factors and extracellular matrix genes in murine ESCs (Supplementary Fig. 6c). Taken together, these data indicate that our tenogenic differentiation protocol produces EGFP-positive cells with tenocyte properties derived from iPSCs.

### Tenocyte-like iPSC-derived cells promote the regeneration of injured tendons *in vivo*

Because the iPSC-derived EGFP-positive cells demonstrated tenocyte properties *in vitro*, we sought to confirm whether EGFP-positive tenocyte-like cells could contribute to tendon regeneration *in vivo*. We transected both Achilles tendons in immunocompromised mice (n = 3), and transplanted FACS-sorted EGFP-positive cells mixed with atelocollagen into injured Achilles tendons on the left, while atelocollagen alone (control treatment) on the right, and analysed the extent of regeneration 4 wk after transplantation. Control contralateral hindlimbs treated with atelocollagen alone had more severe skin ulcers than those transplanted with EGFP-positive cells, which were nearly normal in appearance (Supplementary Fig. 7a).

**Figure 4:**
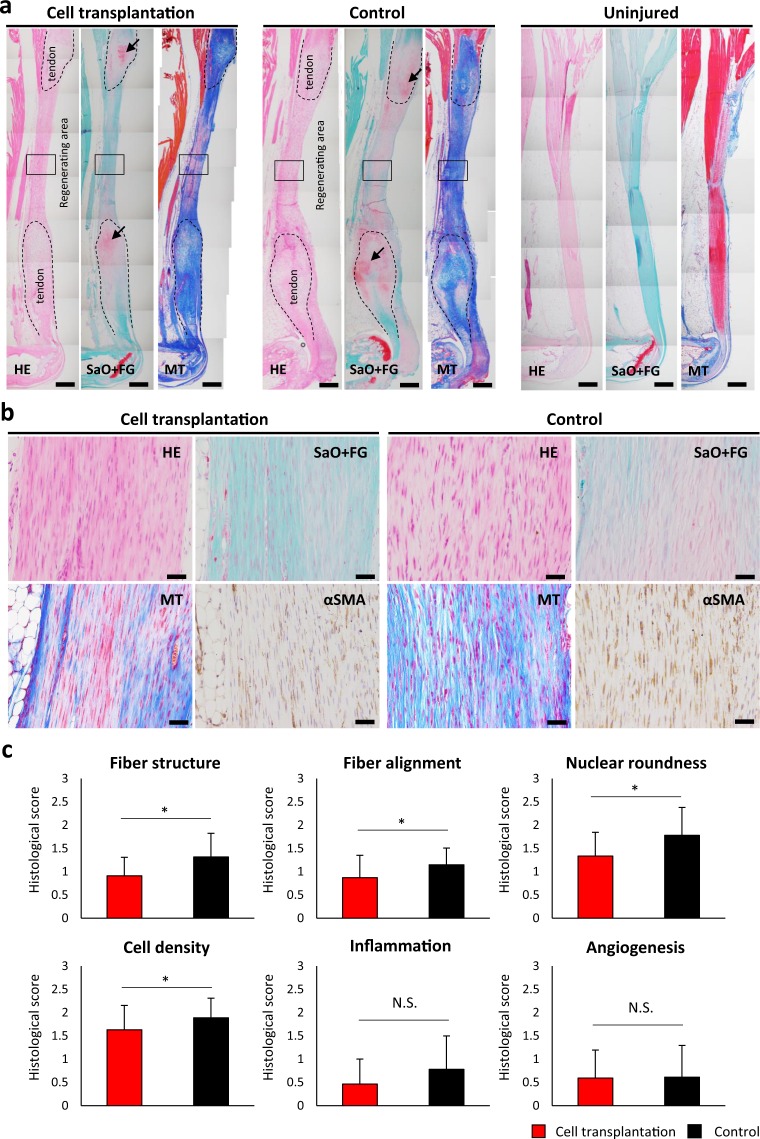
iPSC-derived *Scx-EGFP*-positive cells contribute to tendon regeneration. (**a**) Images of the hindlimbs were acquired 4 wk after transection of Achilles tendons. Both ends of injured Achilles tendons are marked by dotted line, and regenerating areas are in the gap between tendons. The left panel of each group shows tendons transplanted with cells and atelocollagen, the middle panel shows control treatment (atelocollagen alone), and the right panel shows uninjured tendons. Regenerating areas of cell-transplanted tendons are more similar to uninjured tendons than those of the controls. HE, hematoxylin/eosin; SaO + FG, safranin O and fast Green; MT, Masson trichrome. Scale bars, 400 μm. Areas in black squares are shown at higher magnification in Fig. 4b. Black arrows indicate chondrometaplastic lesions on tendon stumps. (**b**) Micrographs of regenerating areas magnified from black squares in Fig. S8A. Cell-transplanted tendons contained well-aligned, spindle-shaped cells (HE, SaO + FG, MT) and fewer myofibroblasts (αSMA) than those of the controls. Scale bar, 50 μm. (**c**) Histology scores for tendon healing. Three sections from each sample were randomly selected and three different fields in each section were analysed by two blinded observers (×40) (mice; n = 3, sections; n = 9, and fields; n = 27). Cell transplantation produced improved fiber structure, fiber alignment, nuclear roundness, and cell density. The score in each category is shown by mean ± SD. Student’s *t*-test was used for statistical analysis. Asterisks indicate statistical significance (*P* < 0.01). N.S., not significant.

The histological analysis showed regenerative tendon formation between Achilles tendon stumps with chondrometaplastic lesions, consistent with typical healing in adult mice (Supplementary Fig. 4b); moreover, no tumour formation was observed (Fig. 4a,b). The hindlimbs with EGFP-positive cell transplantation exhibited well-aligned collagenous fibre formation with spindle-shaped nuclei (Fig. 4b) and significantly better tendon regeneration than controls in terms of fiber structure, fiber arrangement, nuclear roundness, and cell density (Fig. 4c). Immunohistochemistry showed that transplanted cells expressing mCherry were present within regenerative tissue between the tendon stumps (mean 4.7% of total cells, data not shown), and these cells also expressed EGFP and Tnmd (Supplementary Fig. 7b,c). These data demonstrate that EGFP-expressing tenocyte-like cells derived from murine iPSCs promote the regeneration of injured tendons.

### Tendon healing is promoted by paracrine mechanism via transplantation of tenocyte-like iPSC-derived cells

**Figure 5:**
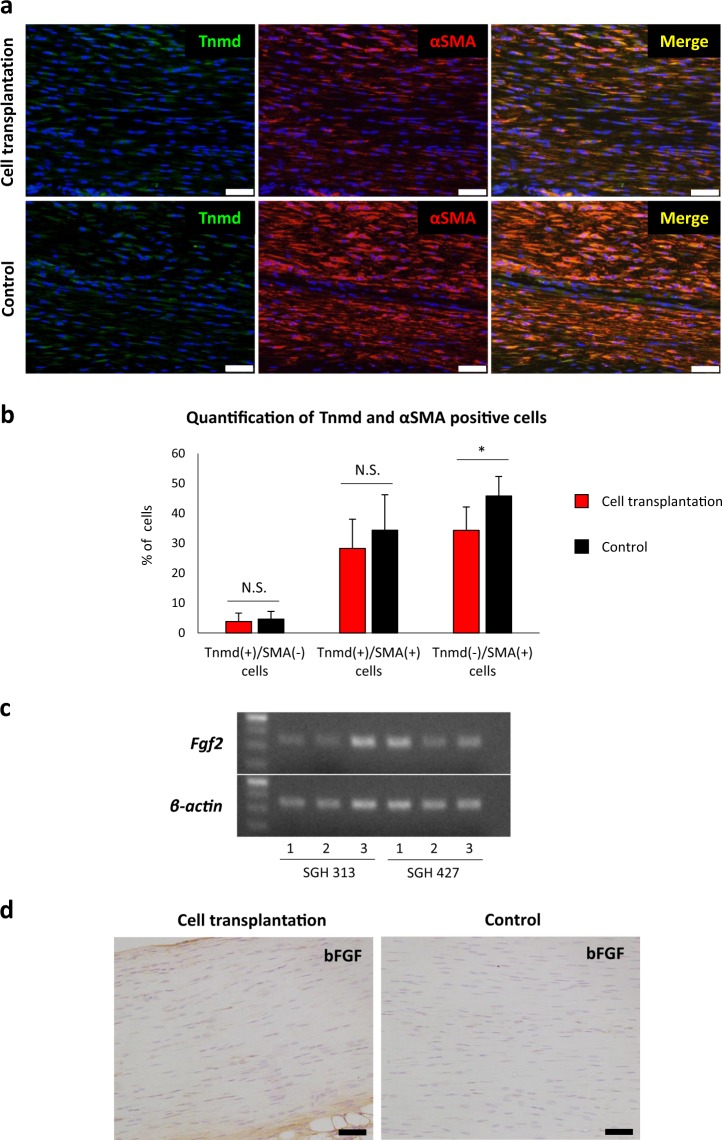
Transplantation of iPSC-derived tenocyte-like cells reduce scar formation around injured Achilles tendons via paracrine effect. (**a**) αSMA and Tnmd expression in regenerating tendons following cell transplantation and control treatment (same area in Fig. 4b). Scale bar, 50 μm. (**b**) Quantitation of αSMA- and Tnmd-positive cells in regenerative tendons. Three different fields in each sample (n = 3 mice) (Supplementary Fig. 7a) were analysed. Mann–Whitney U test was used to compare between the experimental and control groups. Asterisks indicate statistical significance (*P* < 0.05). N.S., not significant. (**c**) RT-PCR shows *Fgf2* expression in SGH 313- and 427-derived EGFP positive tenogenic cells that were sorted by flow cytometry on day 20. Three independent experimental samples in each iPSC line were used. The number of PCR cycles was 35 for *Fgf2* and 30 for *β-actin*. (**d**) Immunohistological detection of bFGF in the regenerating tendons 4 wks after transection. Regenerating tendon in cell transplantation shows bFGF expression, whereas that in control shows little expression. Scale bar, 50 μm.

Finally, we tried to analyse the mechanism of tendon healing via the transplantation of iPSC-derived tenocyte-like cells. A previous study has shown that tendon midsubstance contains some αSMA-positive tenocytes^33^. We detected both Tnmd^+^/αSMA^+^ cells and Tnmd^+^/αSMA^−^ cells in regenerating tendons. Considering that both cell types are tenocytes, no significant differences in their ratios in regenerative tendons were observed (Fig. 5a,b). Indeed, regenerative tendons in mice with cell transplantation had significantly fewer Tnmd^−^/αSMA^+^ cells (myofibroblasts) than those with control treatment, indicating that cell transplantation reduced scar formation (Figs. 4b, 5a,b).

Notably, a previous study demonstrated that cell transplantation therapy for myocardial infarction limits myofibroblast activation and differentiation through basic fibroblast growth factor (bFGF)-mediated paracrine effect resulted in reduced scar formation^34^. Interestingly, consistent with that, iPSC-derived tenocyte-like cells expressed *Fgf2*, and regenerating tendons with cell transplantation in our experiments showed higher bFGF expression than that in tendons with control treatment (Fig. 5c,d), suggesting anti-scarring effect by bFGF-mediated paracrine mechanism via tenogenic cell transplantation. Together, these results demonstrate that EGFP-expressing tenocyte-like cells derived from murine iPSCs promote the regeneration of injured tendons by reducing scar formation via a paracrine mechanism.

## Discussion

Stem-cell therapies are promising regenerative medicine strategies for multiple musculoskeletal disorders^35–37^. In this study, we established a mouse model expressing a *Scx-EGFP* tendon-specific reporter and derived iPSCs with the reporter. Reporter-based differentiation from pluripotent stem cells has been a useful strategy for the development of differentiation protocols^14,38,39^. By mimicking embryonic tendon development and differentiation, which involve paraxial mesoderm, somite, syndetome, and tendon formation, we developed a new, stepwise tendon differentiation protocol, taking advantage of the *Scx-EGFP* reporter as a marker for successful differentiation.

Activin A and Wnt signal activation is known to be required for the differentiation of murine iPSCs toward early mesendoderm and paraxial mesodermal linage^40^. In this study, Activin A and Wnt3a treatment for EBs induced mesendoderm differentiation expressing *Gsc*, followed by bFGF treatment induced mesoderm differentiation with increased expression of paraxial mesoderm and somite markers (*Tcf15*, *Nkx3.2*, and *Meox1*), and decreased the expression of endoderm marker (*Foxa2*)^31,32^. Upon further induction of differentiation using bFGF and TGF-β1 that are required for tendon development from syndetome^19,29^, the EGFP-positive cells derived from murine iPSCs expressed the tenocyte markers *Scx*, *Mkx*, and *Tnmd*, suggesting robust tenocyte differentiation. Moreover, these cells produced well-aligned collagenous extracellular matrix upon transplantation into injured Achilles tendons and improved histological scores of injured tendons compared with those of the control treatment. It is noteworthy that our study not only provides a new tenogenic differentiation protocol and isolation method, but also demonstrates the efficacy in the treatment of tendon injuries in an animal model.

The therapeutic mechanism of cell therapy is mainly direct ECM production form transplanted cells and paracrine effects via transplanted cells, including angiogenesis, anti-apoptosis, anti-oxidation, anti-scarring, cell migration/stimulation, and immunomodulation^41,42^. Previous studies showed that transplanted tenocytes survived for 4–8 wk and promoted regeneration of injured tendon by producing tendon matrix, on the contrary, a large number of host-derived local cells were also distributed in the regenerating area^18,43^. Consistent with those results, iPSC-derived tenocyte-like cells labelled with mCherry remained viable in regenerating tendons at 4 wk after transplantation. However, only a small percentage (4.7%) of mCherry-positive transplanted cells existed in regenerating area and a large percentage of mCherry-negative cells were observed at implantation sites, suggesting not direct ECM formation via transplanted cells but some paracrine effects could be responsible for tendon regeneration.

Indeed, regenerating tissue in mice with tenocyte-like cell transplantation contained fewer myofibroblasts expressing αSMA than in mice with control treatment, indicating reduced scar formation. Of note, bFGF and HGF are known to possess anti-scar effect^34,44,45^. In the present study, iPSC-derived tenocyte-like cells expressed *Fgf2* and regenerative tendon with cell transplantation expressed bFGF compared with that with control treatment. We propose that bFGF-mediated paracrine effect that limits myofibroblast-induced scar formation can be one of the possible mechanisms of tendon healing via cell transplantation.

For further development of iPSC-derived tenogenic cell transplantation therapy, some issues remain to be solved. First is improvement in the induction efficiency of EGFP-positive tenocyte-like cells. Reporter-based differentiation strategy is a powerful tool. Kanke *et al*.^14^ induced osteoblasts from murine iPSCs and ESCs using a *Col1a1-GFP* reporter, with 45% efficiency. Diekman *et al*.^46^ induced chondrocytes from murine iPSCs with a *Col2-GFP* reporter, with 10.3% efficiency. Although the induction efficiency of target cells may depend on the cell type, in our protocol, the overall induction efficiency of EGFP-positive tenocyte-like cells was 11%. Actually, in our experiments, approximately 4 × 10^4^ cells were required to assist the regeneration of a 2-mm tendon injury in mice. Obviously, considerably higher number of cells would be required for human tendon injuries. We need to further optimise our protocol to achieve higher induction efficiency. Second is the method of cell transplantation. For our transplantation method, we used an atelocollagen matrix. Recently, several tissue-engineered constructs have been developed for tendon regeneration^47,48^. An enhanced regenerative effect of tendon-cell transplantation when combined with tendon-derived decellularised extracellular matrix has been reported^49^. As reported by Zhang *et al*.^18^, the combination of induced tenocyte-like cells and these tissue-engineered constructs may have greater therapeutic potential than cell transplantation alone. Indeed, our iPSC-derived tenocyte-like cells promoted the regeneration of injured tendons but did not completely restore a state similar to that of uninjured, healthy tendons. In the future, we can apply our tenocyte differentiation protocol in combination with tissue-engineered constructs to obtain more efficient regeneration.

In summary, we have developed a tenocyte induction protocol for murine iPSCs with a *Scx-EGFP* tendon-specific reporter system. Histology and immunohistochemistry demonstrated that murine iPSC-derived tenocyte-like cells promoted significant improvements in the regeneration of injured tendons via a paracrine mechanism in mice. Pluripotent stem cell-based cell therapy using this tenogenic differentiation protocol may be an effective therapeutic approach for tendon injuries.

## Methods

### Animal experiments

All mouse experiments were approved by the Gifu University and CiRA Animal Experiment Committee (28–19, 28–65, 28–89 and H30–077), and were in compliance with the Animal Research: Reporting *in Vivo* Experiments guidelines.

### Embryonic stem cell (ESC) targeting and generation of *Scx-EGFP* mice

For *Scx-EGFP* knock-in, the Red/ET BAC recombination system was used to introduce an *IRES-EGFP-pA-rox-PGK-EM7-BsdR-pA-rox* cassette into the 3′ UTR of *Scx* BAC (RP23-415D19) to generate targeting vectors. The resultant constructs were electroporated into V6.5 ESCs. ESCs were cultured with ES medium containing 15 µg/mL Blasticidin S (Bsd, Funakoshi, Tokyo, Japan). Drug-resistant colonies were selected for expansion. Correctly targeted ES clones were confirmed by PCR and southern blotting. Chimeric mice were generated from the ESC clones and mated with wild-type C57BL/6 mice. To remove the drug selection cassette, *Scx-EGFP* mice were crossed with Dre-expressing mice (Tg(CAG-dre)1Afst)^28^. *Scx-EGFP* mice without drug selection cassettes were backcrossed with C57BL/6 mice for at least six generations before being used in experiments.

### *In vivo* experiments

For teratoma generation, 3 × 10^6^ iPSCs were transplanted subcutaneously into BALB/c *nu/nu* mice (female) purchased from CLEA Japan (https://www.clea-japan.com/). Teratomas were observed after 3–4 wk. Immunofluorescence of tendons from *Scx-EGFP* mice, teratomas, and transplanted EGFP-positive cells derived from iPSCs was confirmed by fluorescence microscopy.

### iPSC induction and maintenance

iPSC induction was performed using the retroviral vectors pMXs-hOCT3/4, pMXs-hSOX2, pMXs-hKLF4, and pMXs-Hu-L-MYC (Addgene). After transduction with the reprogramming factors, *Scx-EGFP* homozygous mice-derived ear tip fibroblasts were cultured in ESC medium supplemented with 1000 U/mL human recombinant LIF (FUJIFILM Wako Pure Chemical Corporation, Osaka, Japan), 0.11 mM 2-Mercaptoethanol (Thermo Fisher Scientific, Tokyo, Japan) and 50 µg/mL L-ascorbic acid (Sigma-Aldrich, Tokyo, Japan). The established iPSCs (SGH iPSCs) were maintained in ESC medium supplemented with 1000 U/mL human recombinant LIF and 0.11 mM 2-Mercaptoethanol.

### Fluorescent labelling of iPSCs

A pBRY-nuclear mCherry-IRES-PURO construct (Addgene) was electroporated into SGH iPSC clones 3–1 and 4–2. Cells were cultured with ES medium containing 1 μg/mL puromycin (Thermo Fisher Scientific), and puromycin-resistant colonies were selected and expanded. Ubiquitously and brightly labelled clones SGH 313 and SGH 427 were used for tenocyte differentiation experiments.

### RT-PCR and real-time quantitative PCR

RNA was extracted using the RNeasy Plus Mini Kit (Qiagen). Up to 1 µg of RNA was used for reverse transcription into cDNA. RT-PCR was performed using KOD-Fx-Neo (Toyobo, Osaka, Japan) and AmpliTaq Gold 360 (Thermo Fisher Scientific), and real-time quantitative PCR was performed using Premix Ex Taq (Perfect Real Time) (Takara, Kusatsu, Japan). Transcript levels were normalised to β-actin. PCR primer sequences are provided in Supplementary Table S1.

### DNA copy number analysis

Genomic DNA was extracted using the QIAamp DNA Mini Kit (Qiagen). Real-time quantitative PCR was performed using Premix Ex Taq (Perfect Real Time) (Takara, Kusatsu, Japan). Primers were designed for common sequences between human *OCT3/4*, *SOX2*, *KLF4*, and *MYCL* and mouse *Oct3/4*, *Sox2*, *Klf4*, and *Mycl*. Total copy numbers were normalised to endogenous mouse *Pecam1* and endogenous DNA copy number of control (*Scx-EGFP* mice-derived fibroblasts) was set to 2. Copy numbers of exogenous *OCT3/4*, *SOX2*, *KLF4*, and *MYCL* in SGH 313 and 427 were calculated as follows: exogenous copy number = total copy number - 2 (endogenous copy number), respectively. PCR primer sequences are provided in Supplementary Table S1.

### Immunocytochemistry

Cultured cells were washed with PBS and fixed with 2% paraformaldehyde for 15 min at room temperature. For immunocytochemistry, the primary antibody used was anti-tenomodulin (Abcam; dilution 1:200), and the cells were incubated overnight at 4 °C. The CF350 (Biotium) secondary antibody was subsequently added to cells, which were analysed by fluorescence microscopy (IX83, Olympus).

### Histology and immunohistochemistry

All tissue samples were fixed with 4% paraformaldehyde overnight, decalcified with pH 7.2 EDTA buffer (G-Chelate Mild, GenoStuff, Tokyo, Japan) for 10 d at 4 °C, and embedded in paraffin. The samples were cut into 3–4-μm-thick sections. Hematoxylin and eosin, safranin O/fast Green, and Masson trichrome staining were applied using standard protocols. For immunohistochemistry, the antibodies used were anti-GFP (EPR14104) (Abcam; dilution 1:100), anti-GFP (4B10) (Cell Signaling Technology; dilution 1:200), anti-RFP (5F8) (Chromotek; dilution 1:200), anti-tenomodulin (Thermo Fisher Scientific; dilution 1:150), anti-αSMA (1A4) (Abcam; dilution 1:200), and anti-bFGF (Bioss; dilution 1:200). For DAB staining, the secondary antibody used was Dako EnVision (Dako Japan Inc., Kyoto, Japan); stained cells were analysed by microscopy (BX51, Olympus). For immunofluorescence, the secondary antibodies were conjugated with Alexa Fluor 488 and Alexa Fluor 594 (Thermo Fisher Scientific); stained cells were analysed by fluorescence microscopy (IX83, Olympus).

### *In vitro* differentiation of iPSCs and ESCs into tenocytes

Suspensions of 5000 iPSCs and ESCs (V6.5)/well were cultured in a 96-well plate (Nunclon Sphere, Thermo Fisher Scientific) with differentiation medium (DMEM high glucose, 10% FBS, 100 U/mL penicillin/100 µg/mL streptomycin, 2 mM L-glutamine). On day 2, Wnt3a (R&D Systems, Minneapolis, USA) and Activin A (Peprotech, Rocky Hill, USA) were added at final concentrations of 25 and 9 ng/mL, respectively; on day 3, EBs were cultured in differentiation medium supplemented with bFGF (FUJIFILM Wako Pure Chemical Corporation, Osaka, Japan) at a final concentration of 10 ng/mL for 2 d. On day 5, EBs were harvested and dissociated to single cells with TrypLE Express (Thermo Fisher Scientific). Cells were cultured in a collagen-coated six-well plate (Thermo Fisher Scientific) in differentiation medium supplemented with 1% insulin-transferrin selenium (ITS; Thermo Fisher Scientific), 10 ng/mL TGF-β1 (Cell Signaling Technology, Tokyo, Japan), and 10 ng/mL bFGF. Media were changed every other day. Cell sorting by FACS (Aria II, BD) was performed on day 20. Sorted cells were used for real-time quantitative RT-PCR and transplantation experiments.

### Cell transplantation

FACS-sorted, iPSC-derived, EGFP-positive cells were mixed with pepsin-solubilised collagen (Nippi, Tokyo, Japan) and transplanted (n = 3) into 6-wk-old NOD-SCID female mice purchased from Charles River, Japan. Under anaesthesia (Vetorphale, Dorbene, and Dormicum), approximately 2-mm wide resections were made in both Achilles tendons. On one side, 10 µL of 4 × 10^6^ cells/mL suspension was transplanted into the gap between the tendon stumps. On the contralateral side, pepsin-solubilised collagen alone was injected into the gap. Four weeks after transplantation, the mice were euthanised and regenerating tendons were histologically evaluated.

### Quantitative evaluation of tendon regeneration

To evaluate regenerative efficiency *in vivo*, we histologically analysed fiber alignment, fiber structure, nuclear roundness, cell density, inflammation, and angiogenesis according to a grading system utilised in previous studies^43,50,51^. These six parameters were quantified by two blinded observers using 0–3 grading scores: 0 (normal), 1 (slightly abnormal), 2 (moderately abnormal), and 3 (severely abnormal).

### Statistics

Data from real-time quantitative RT-PCR, quantitative grading of tendon healing, and percentages of αSMA-, Tnmd-, and mCherry-positive cells in regenerating tendons are presented as mean ± SD. Student’s *t*, Mann–Whitney U, and ANOVA tests (Kruskal–Wallis) were used for statistical analysis. Differences were considered statistically significant at *P* < 0.05.

## Supplementary information

Supplementary information.
