# Supplementary material for: Induced pluripotent stem cell-derived tenocyte-like cells promote the regeneration of injured tendons in mice
Source: Sci Rep. 2020 Mar 4;10:3992. doi: 10.1038/s41598-020-61063-6 (PMC7055210; doi:10.1038/s41598-020-61063-6)
Supplement: Supplementary file 1 — Supplementary information. [file 41598_2020_61063_MOESM1_ESM.pdf]

Induced pluripotent stem cell-derived tenocyte-like cells promote the regeneration of injured tendons in mice

Authors: Shingo Komura[1,3,*] , Takashi Satake[1] , Atsushi Goto[1] , Hitomi Aoki[2] , Hirofumi Shibata[3] , Kenji Ito[3,4] ,Akihiro Hirakawa[1] , Yasuhiro Yamada[3,4] , Haruhiko Akiyama[1]

Affiliations:
[1] Department of Orthopaedic Surgery, Gifu University Graduate School of Medicine, Gifu 501-1194, Japan
[2] Department of Tissue and Organ Development, Regeneration, and Advanced Medical Science, Gifu University Graduate School of Medicine, Gifu 501-1194, Japan
[3] Laboratory of Stem Cell Oncology, Department of Life Science Frontiers, Center for iPS Cell Research and Application (CiRA), Kyoto University, Kyoto 606-8507, Japan
[4] Division of Stem Cell Pathology, Center for Experimental Medicine and Systems Biology, Institute of Medical Science, University of Tokyo, Tokyo 108-8639, Japan

***Correspondence**
Shingo Komura, MD, PhD
Department of Orthopaedic Surgery, Gifu University Graduate School of Medicine
1-1 Yanagido, Gifu 501-1194, Japan.
Email: s_komura@gifu-u.ac.jp
Tel: +81-58-230-6333, Fax: +81-58-230-6334

Supplemental Information

Legends to Supplemental Figures (Supplemental Fig. 1-8)

Legend to Supplemental Table 1

Supplementary Fig. 1

***Scx-EGFP* reporter system and ES cell targeting**

(a) Schematic of *Scx* targeting. *IRES-EGFP* was inserted into the 3′ UTR of the *Scx* locus. IRES, internal ribosome entry site; pA, poly(A) sequence; Bsd^R, blasticidin resistance gene; DT-A, diphtheria toxin A; H, HindIII; S, SspI; A, AgeI.

(b) Southern blot of the Bsd-resistant clone using 5′ and 3′ external probes. The sequences of the primers for the 5′ external probe were as follows: forward, 5′-TGAGAAGGGATCGGGAAACATG-3′; reverse 5′-AGATGAGCTCGACCAGTTTCTG-3′. The sequences of primers for the 3′ external probe were as follows: forward, 5′- ACAATGCGGGTAGTGTTCAC-3′; reverse, 5′- CACAAAACAGGCACCCTTTG-3′. Homologous recombination was confirmed in clone #8.

Supplementary Fig. 2

Detection of EGFP in tissues of *Scx-EGFP* mice

Immunohistochemistry of *Scx-EGFP* and wild-type (WT) mice using the anti-GFP antibody. The intermuscular tendon and anulus fibrosus of the intervertebral disc are EGFP-positive (black arrows), whereas muscles around the tendon and multiple organs

are EGFP-negative. Scale bar, 50 µm.

Supplementary Fig. 3

Generation of homozygous *Scx-EGFP* reporter mice

(a) Schematic of deletion of the *PGK-Bsd^R-pA* cassette by Dre recombinase. *Scx-EGFP* heterozygous mice were crossed with Tg(CAG-dre)1Afst mice. Right, PCR showing deletion of the drug-selection cassette. The sequences of the primers used were as follows: forward, 5′-TATGCTGGCTGCCATGAACA-3′; reverse, 5′-ACTAGTGGCATCACCTCTTGG-3′.

(b) Image of 3-wk-old *Scx-EGFP* mice. No differences were observed between homozygous (left), heterozygous (middle), and wild-type (right).

(c) Fluorescence images of *Scx-EGFP* homozygous (left), heterozygous (middle), and control wild-type (right) mouse knees. The *Scx-EGFP* homozygous mouse knees exhibited a higher signal than the heterozygous mouse knees, indicating bi-allelic expression of *Scx* knock-in. All tendons were imaged under identical settings. P, patella; asterisk, patellar tendon; arrow, collateral ligament.

Supplementary Fig. 4

Superior recovery from tendon injury in neonatal mice contrasted with scarring in adult mice

(a) Recovery of neonatal and adult mice from Achilles tendon transection. We transected Achilles tendons when the neonatal mice were 7-d-old and adult mice were 4-d-old, and analysed at 10 and 30 d after transection. Upper and lower images show neonatal and adult mice tendons, respectively. Regenerative areas between the tendon stumps are indicated by white dotted boxes. At 10 days after transection, EGFP-negative tissue was connected to the tendon stumps in both neonatal and adult mice (left panels). However, 30 d after transection, slightly EGFP-positive tissue was connected to the tendon stumps in adult mice, whereas strongly EGFP-positive tissue connected them in neonatal mice (middle panels). Uninjured control tendons from neonatal and adult mice are shown in the right panels.

(b) Histology and immunohistochemistry of Achilles tendons 30 d following tendon transection. The left group of images shows neonatal and the right shows adult specimens. Neonatal mice had physiological tendon healing with less scar tissue expressing αSMA. Conversely, adult mice had scarring between the tendon stumps and chondrometaplasia was observed on the tendon stump (black arrow). SaO+FG; safranin O and fast green, MT; Masson trichrome.

Supplementary Fig. 5

Generation of iPSCs and teratomas

(a) Phase-contrast micrographs of *Scx-EGFP* ear-tip fibroblasts and their derived iPSCs. Scale bar, 100 μm.

(b) RT-PCR showed no obvious exogenous 4-factor expression in SGH iPSCs. Negative control (Neg.) is *Scx-EGFP* ear-tip fibroblasts and positive control (Pos.) is tip fibroblasts at 6 d after retroviral 4-factor introduction. The number of PCR cycles is shown. Ex; exogenous.

(c) The number of exogenous transgene integration in SGH iPSCs (313 and 427) analysed by real-time PCR. Control DNA was obtained from *Scx-EGFP* mouse fibroblasts. SGH 313 contains 3, 12, 3, and 7 copies of exogenous *OCT3/4*, *SOX2*, *KLF4*, and *MYCL*, and SGH 427 contains 11, 13, 2, and 14 copies of exogenous *OCT3/4*, *SOX2*, *KLF4*, and *MYCL*, respectively. Endogenous genome of *Pecam1* was used as the standard. Data are presented as the mean of three technical replicates.

(d) Micrographs of a teratoma derived from SGH iPSC clone 313 (Left). The teratoma expresses mCherry (right) but not EGFP (middle).

(e) SGH iPSC teratomas containing ectodermal, mesodermal, and endodermal tissues in the subcutaneous tissue of immunocompromised mice. Scale bar, 50 µm.

Supplementary Fig. 6

Gene expression in differentiated tenogenic cells

(a) Mesodermal differentiation by embryoid body (EB) formation. mRNA was extracted from 10–16 EBs generated from SGH 313 and 427 at days 3 and 5, and measured by qRT-PCR. Upregulation of mesendoderm marker (*Gsc*) at day 3 and downregulation of endoderm marker (*Foxa2*) and upregulation of mesoderm marker (*Tcf15*, *Nkx3.2*, and *Meox1*). The mean ± SD (three technical replicates per n; n = 3 biological replicates) is shown. Expression levels on day 0 were set to 1. ANOVA test (Kruskal–Wallis test) was used for statistical analysis. Asterisks indicate statistical significance ($P < 0.05$).

(b) Expression levels of pluripotency-related genes in FACS-sorted EGFP-positive cells 20 d after induction of tenogenic differentiation. Expression was measured by qRT-PCR and is presented as the mean ± SD (three technical replicates per n; n = 3 biological replicates). The expression levels of ESCs were set to 1. Black bars, iPSCs; red bars, FACS-sorted EGFP-positive cells on day 20; and yellow bars,

ESCs. Mann–Whitney U test was used to compare between SGH 313 iPSCs and 313 EGFP-positive cells, and between SGH 427 iPSCs and 427 EGFP-positive cells. Asterisks indicate statistical significance ($P < 0.05$).

(c) Tenogenic marker expression in ESCs. mRNA was extracted from a pool of differentiated cells on days 0, 12, and 19 measured by qRT-PCR, showing upregulation of tenogenic differentiation-related genes on day 19. The mean ± SD (three technical replicates per n; n = 3 biological replicates) is shown. Expression levels on day 0 were set to 1. ANOVA test (Kruskal–Wallis test) was used for statistical analysis. Asterisks indicate statistical significance ($P < 0.05$).

Supplementary Fig. 7

iPSC-derived tenocyte-like cells contribute to tendon regeneration *in vivo*

(a) Hindlimbs 4 wk after transplantation of EGFP-positive cells into transected Achilles tendons. We transected both Achilles tendons in immunocompromised mice and performed transplantation experiment. Left hindlimbs with cell transplantation (EGFP-positive cells mixed with atelocollagen; upper image) and control right hindlimbs without cell transplantation (atelocollagen alone; lower image) are shown. Control hindlimbs had more severe ulceration around the ankles. The cell

transplantation and control experiment was performed with three mice from each group (two representative experiments).

(b) mCherry and EGFP expression in regenerating region following cell transplantation (same area in Fig. 4b). Note the co-expression of mCherry and EGFP (white arrows). Scale bars, 20 μm.

(c) Tendon-specific marker (Tmnd) expression in regenerating region following cell transplantation (same area in Fig. 4b). Note the co-expression of mCherry and Tnmd (white arrows). Scale bars, 20 μm.

Supplementary Fig. 8

This file contains uncropped gel image data

Supplementary Table 1

Primer sequences

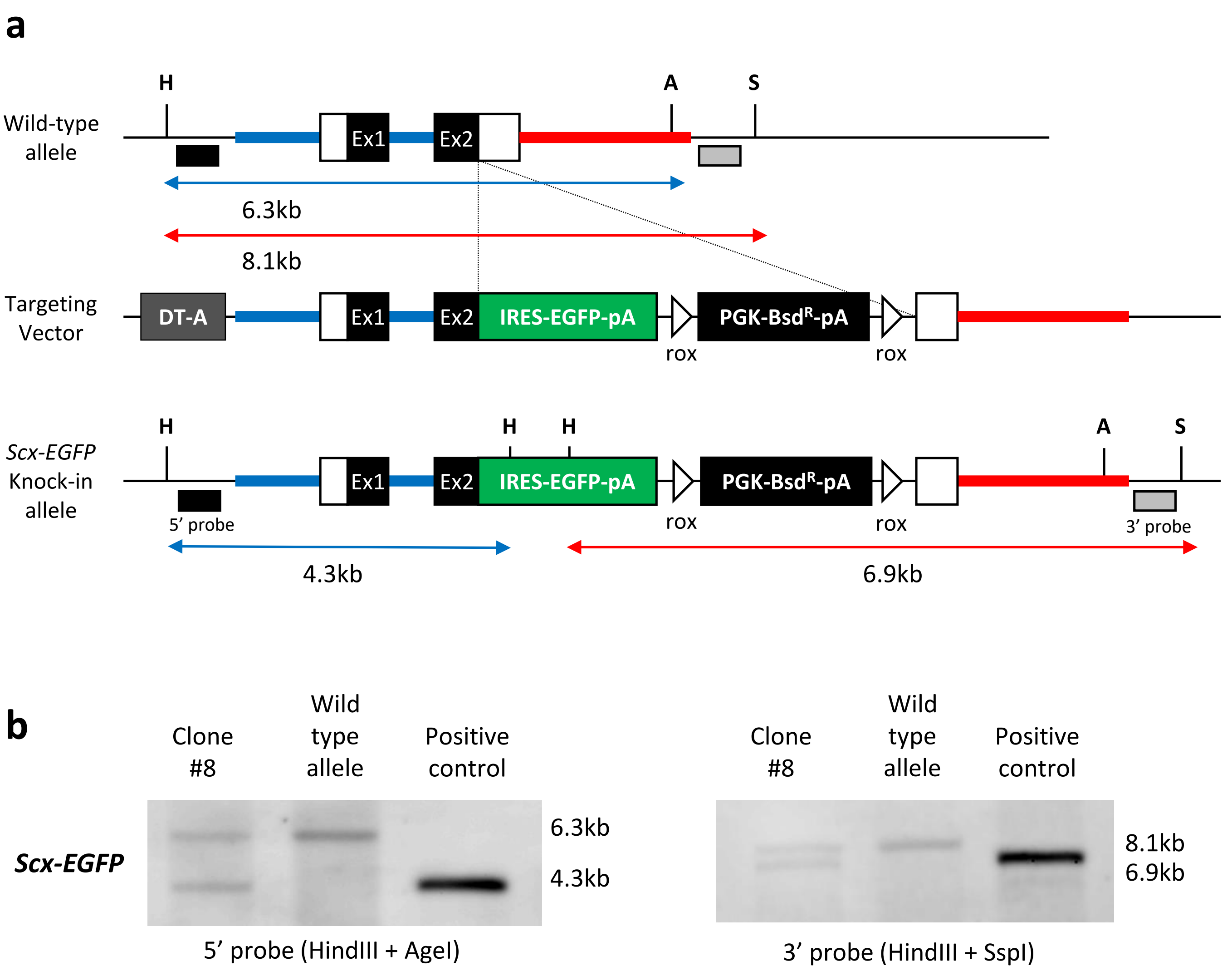
a
H
A
S
Wild-type allele
Ex1
Ex2
6.3kb
8.1kb
Targeting Vector
DT-A
Ex1
Ex2
IRES-EGFP-pA
PGK-Bsd^R-pA
rox
rox
H
H
H
A
S
Scx-EGFP Knock-in allele
Ex1
Ex2
IRES-EGFP-pA
PGK-Bsd^R-pA
5' probe
rox
rox
3' probe
4.3kb
6.9kb
b
Clone #8
Wild type allele
Positive control
Scx-EGFP
6.3kb
4.3kb
5' probe (HindIII + AgeI)
Clone #8
Wild type allele
Positive control
8.1kb
6.9kb
3' probe (HindIII + SspI)

Supplementary Figure 2

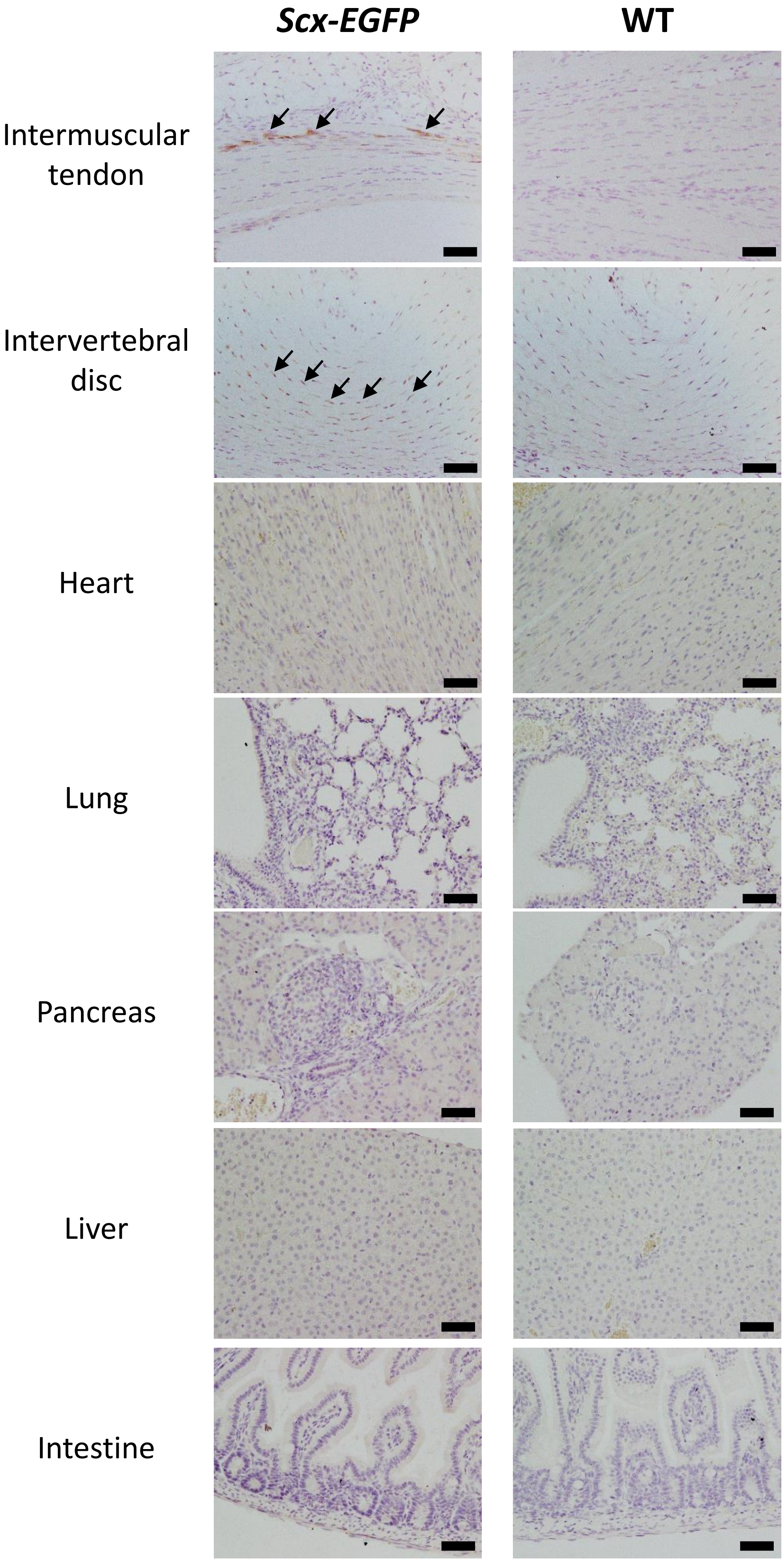

Supplementary Figure 3

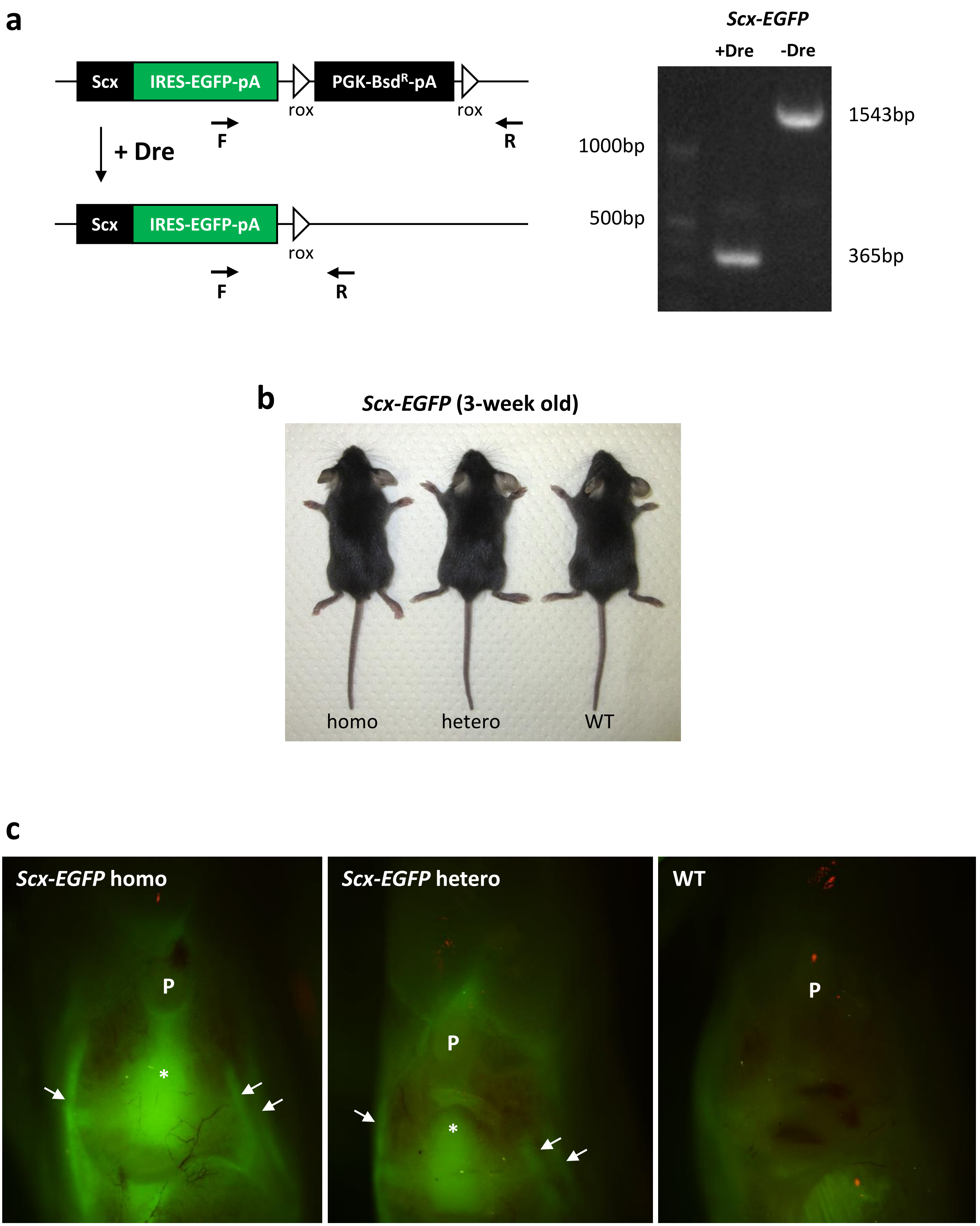

Supplementary Figure 4

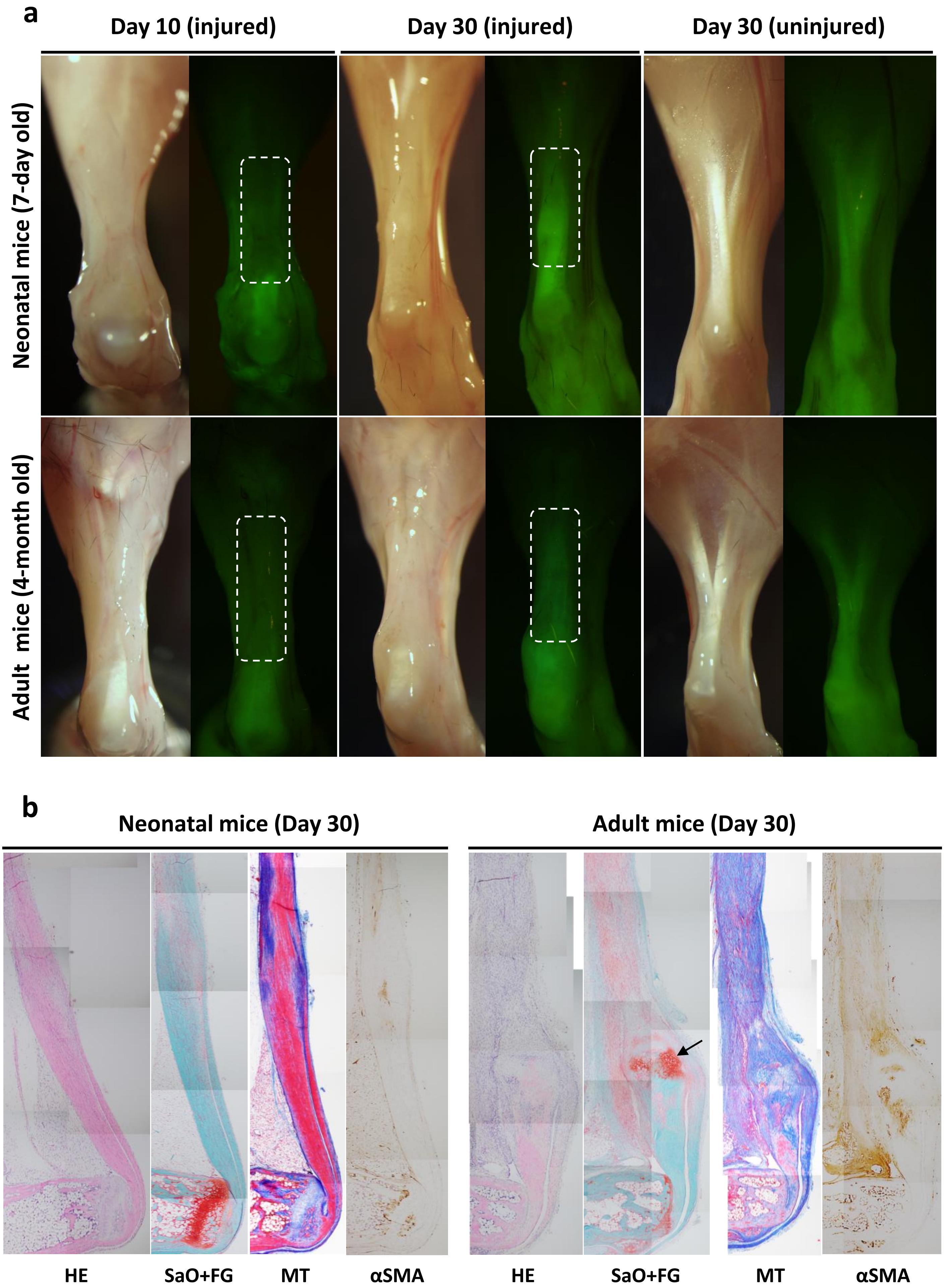

Supplementary Figure 5

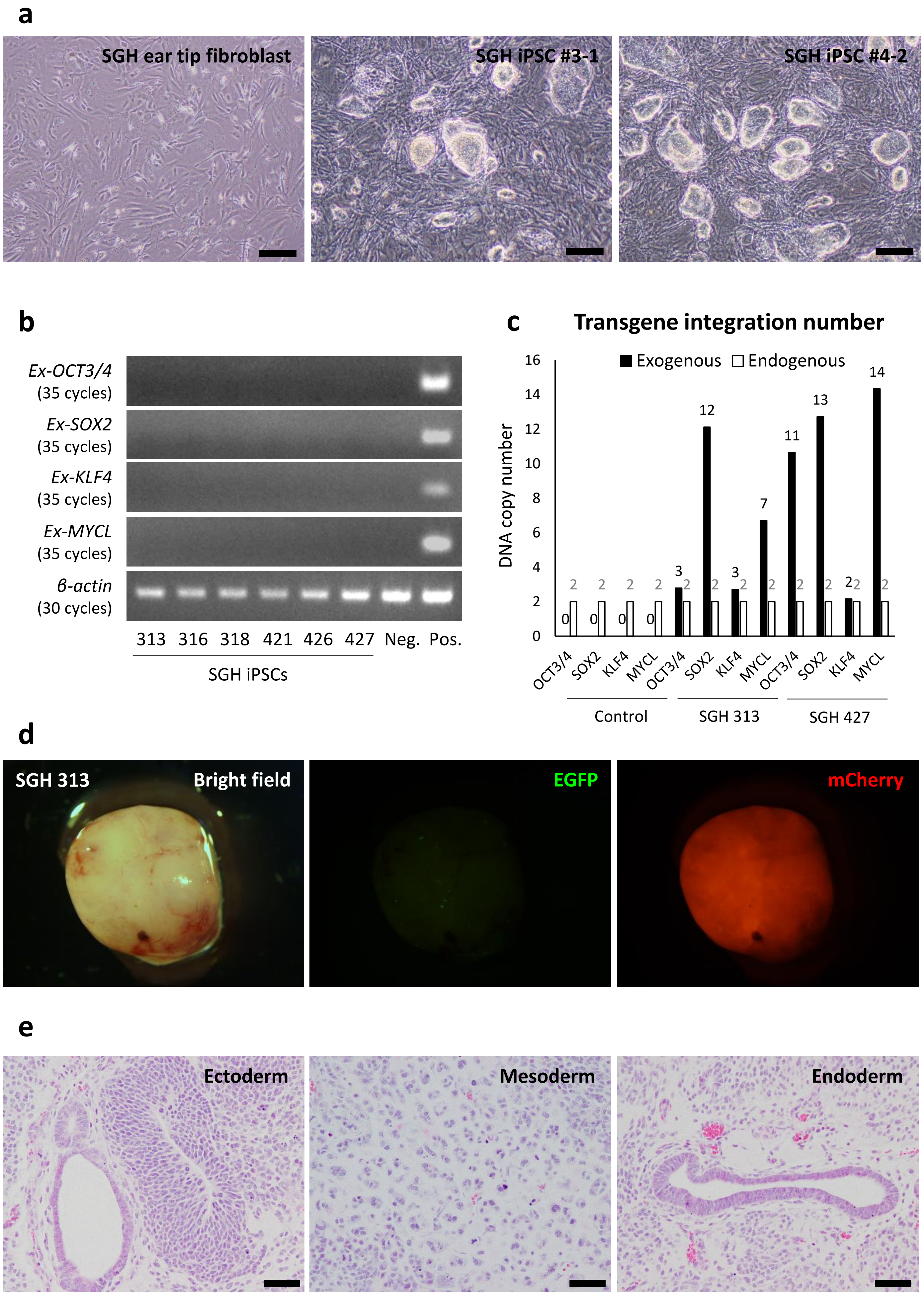

a

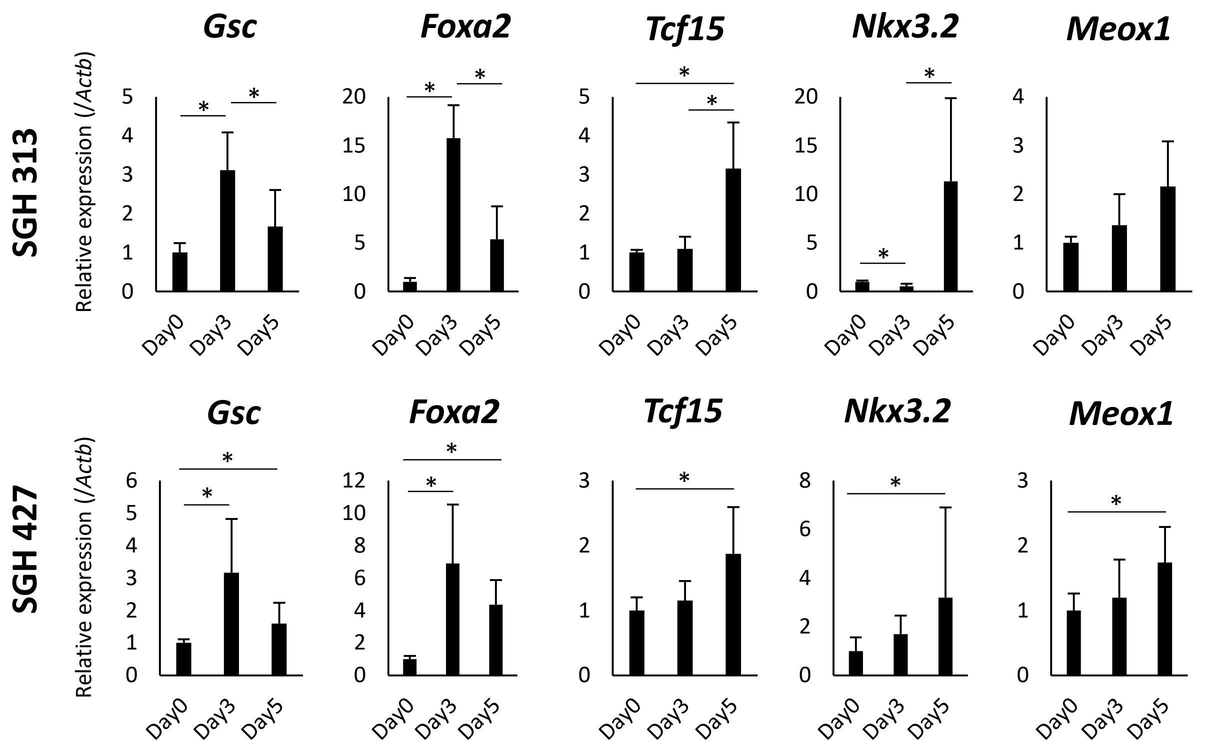
Gsc
Foxa2
Tcf15
Nkx3.2
Meox1
SGH 313
Relative expression (/Actb)
Day0
Day3
Day5
Gsc
Foxa2
Tcf15
Nkx3.2
Meox1
SGH 427
Relative expression (/Actb)
Day0
Day3
Day5

b

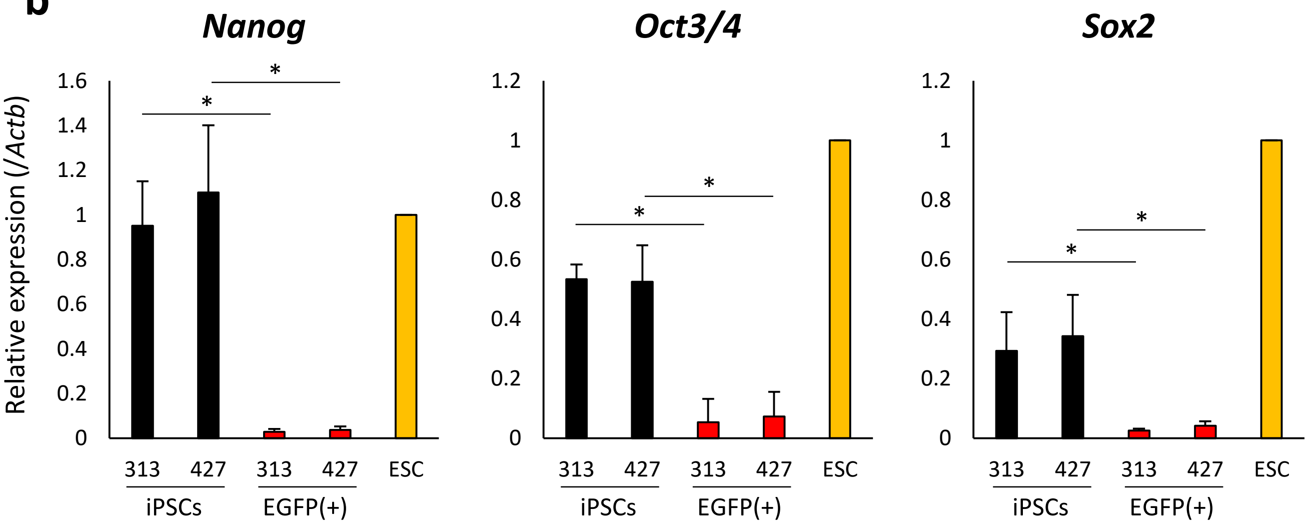
Nanog
Oct3/4
Sox2
Relative expression (/Actb)
313
427
313
427
ESC
iPSCs
EGFP(+)

c

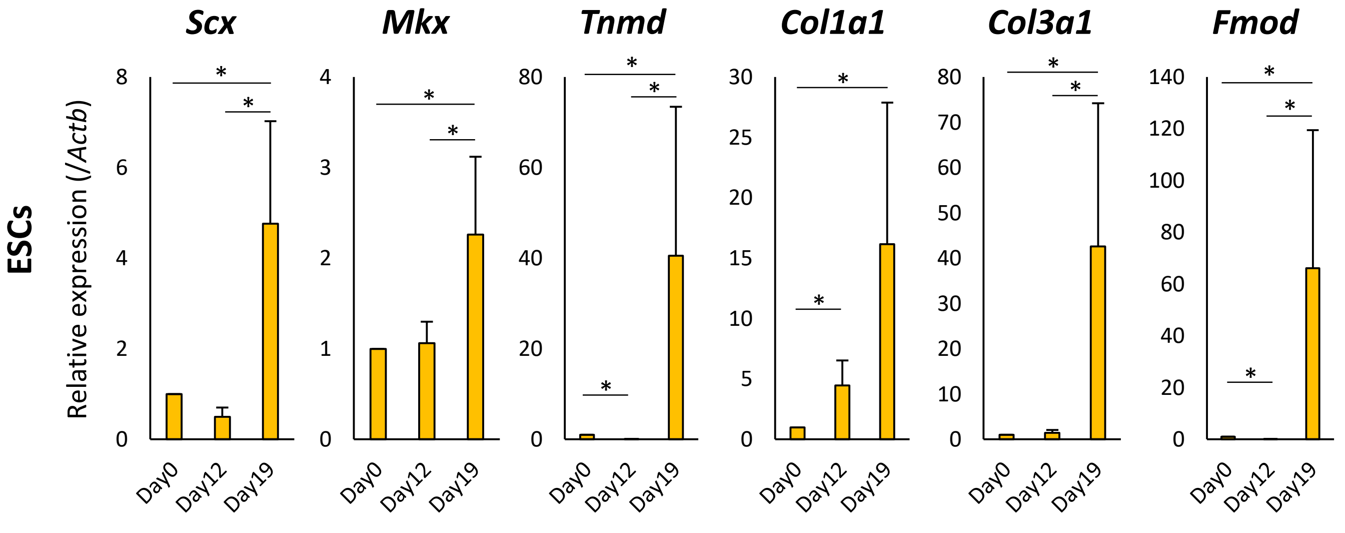
Scx
Mkx
Tnmd
Col1a1
Col3a1
Fmod
ESCs
Relative expression (/Actb)
Day0
Day12
Day19

Supplementary Figure 7

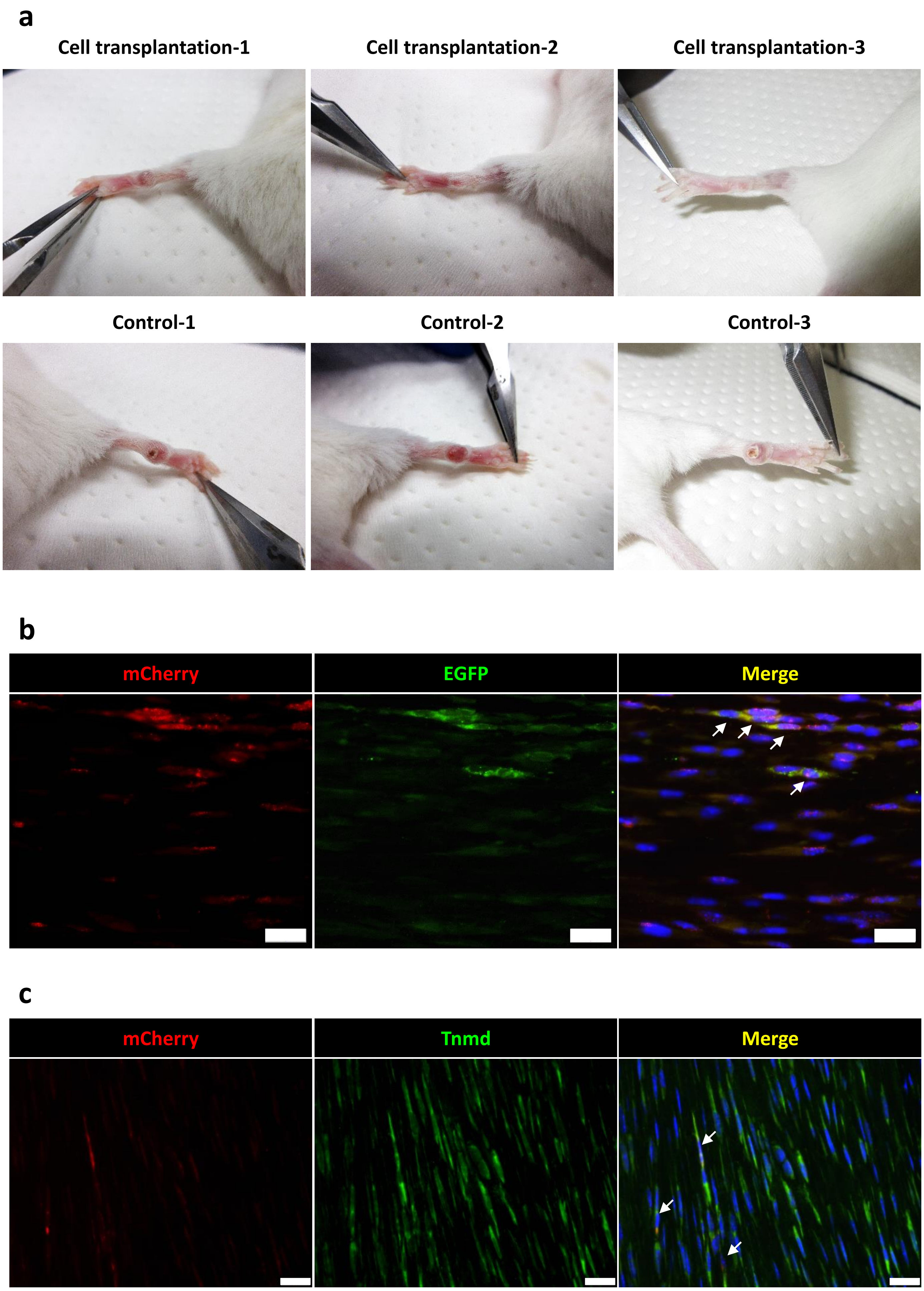

Supplementary Figure 8

Figure 5c

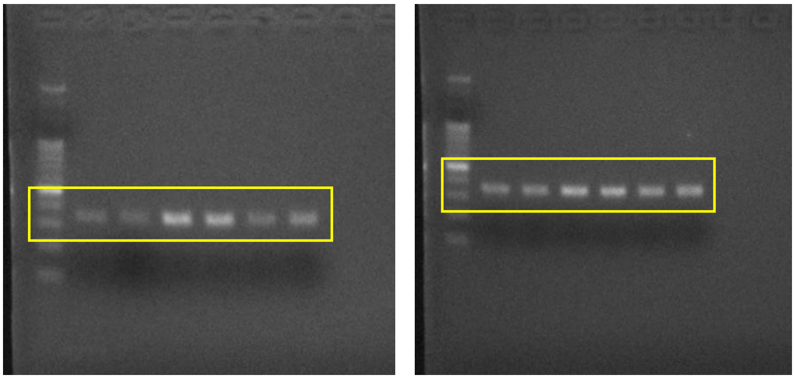

Fgf2 *β-actin*

Supplementary Figure 1b

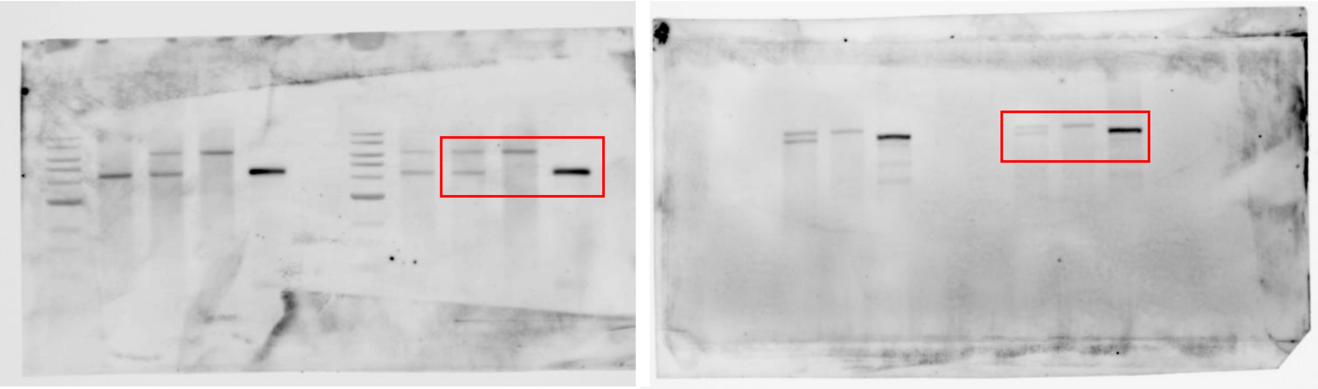

5’ probe (HindIII + AgeI) 3’ probe (HindIII + SspI)

Supplementary Figure 3a

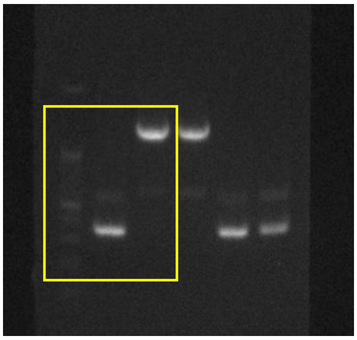

Supplementary Figure 5b

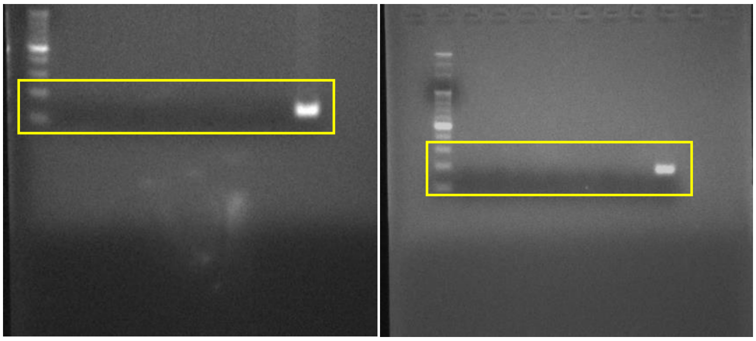

Ex-OCT3/4 *Ex-SOX2*

Supplementary Figure 5b

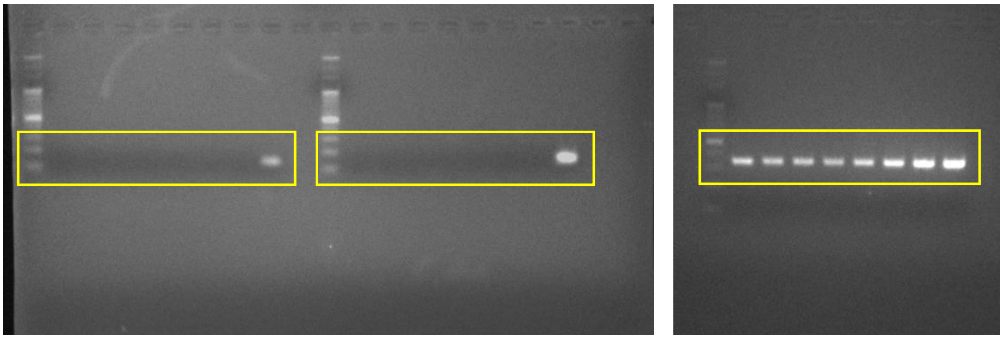

Ex-KLF4 *Ex-MYCL* *β-actin*

Supplementary Table 1

qRT-PCR primer	Forward (5' ⇒ 3')	Revers (5' ⇒ 3')
Nanog	TGCTTACAAGGGTCTGCTACTG	TAGAAGAATCAGGGCTGCCTTG
endogenous Oct3/4	TCCCATGCATTCAAACTGAG	CCACCCCTGTTGTGCTTTTA
endogenous Sox2	ATGGCCCAGCACTACCAGAG	TTTTGCACCCCTCCCAATTCC
Scx	AACACCCAGCCCAAACAGATC	TCTGTCACGGTCTTTGCTCAAC
Mkx	TGAAGGCACCTTTGTCTATCGC	ATGGCTGCATTGATCTCCTTCC
Tnmd	TCCTGTTTTGGGGGAGCAAAC	TTCTCGCCGTTGCTGTAGAAAG
Col1a1	TGGCGGTTATGACTTCAGCTTCCT	GGTCACGAACCACGTTAGCATCAT
Col3a1	CATAATGGGGAACGTGGTCCTC	CTGACCATCTGATCCAGGGTTTC
Fmod	ACAATCAGCTGCAGAAGATCCC	AGAAGTTCATGACGTCCACCAC
Gsc	ATCTTCACCGATGAGCAGCTC	TGGCTCGGCGGTTCTTAAAC
Foxa2	GTGAAGATGGAAGGGCACGAG	ATGTTGCTCACGGAAGAGTAGC
Tcf15	TGCACCTTCTGTCTCAGCAAC	ACACCCCTCACTTTCAAGCAG
Nkx3.2	AAGGACCTGGAGGAGGAAGC	GGGCTAACGCTGTCATCCTC
β-actin	GCCAACCGTGAAAAGATGAC	TCCGGAGTCCATCACAATG

RT-PCR primer	Forward (5' ⇒ 3')	Revers (5' ⇒ 3')
exogeneous hOCT3/4	GAAGCCTTTCCCCCTGTCTC	GACATGGCCTGCCCGGTTATTATT
exogeneous hSOX2	TTCACATGTCCCAGCACTACCAGA	GACATGGCCTGCCCGGTTATTATT
exogeneous hKLF4	CCACCTCGCCTTACACATGAAGA	GACATGGCCTGCCCGGTTATTATT
exogeneous hMYCL	GCAGTTGCAGAAAAGAATTGCA	GACATGGCCTGCCCGGTTATTATT
Fgf2	CGGCTCTACTGCAAGAACG	AGTATGGCCTTCTGTCCAGG
β-actin	GCTACAGCTTCACCACCACA	CTTCTGCATCCTGTCAGCAA

DNA copy number detection primer	Forward (5' ⇒ 3')	Revers (5' ⇒ 3')
hOCT3/4-mOct3/4	TGTAACCGGCGCCAGAAG	CAGTTTGAATGCATGGGAGAGC
hSOX2-mSox2	TGCAGTACAACTCCATGACCAG	TGCGAGTAGGACATGCTGTAG
hKLF4-mKlf4	GAAACCTTACCACTGTGACTGG	CGGTAGTGCCTGGTCAGTTC
hMYCL-mMycl	CTGGAAGAAATTCGAGCTGGTG	AGAAGCCGCTCCACATGC
Pecam1	ATGGAAAGCCTGCCATCATG	TCCTTGTTGTTCAGCATCAC
